# The Silkworm Carboxypeptidase Inhibitor Prevents Gastric Cancer Cells’ Proliferation through the EGF/EGFR Signaling Pathway

**DOI:** 10.3390/ijms24021078

**Published:** 2023-01-05

**Authors:** Junhong Ye, Jifu Li, Ping Zhao

**Affiliations:** 1 State Key Laboratory of Silkworm Genome Biology, Biological Science Research Center, Southwest University, Chongqing 400716, China; 2 College of Sericulture, Textile and Biomass Sciences, Southwest University, Chongqing 400716, China

**Keywords:** silkworm, carboxypeptidase inhibitor, gastric cancer, anti-tumor activity

## Abstract

Gastric cancer is a common malignant tumor originating from the gastric mucosa epithelium. Studies have shown that bioactive substances such as antimicrobial peptides and cantharidin contained in a variety of insects can exert anti-cancer functions; when compared with chemotherapy drugs, these bioactive substances have less toxicity and reduced side effects. Here, we report the first *Bombyx mori* carboxypeptidase inhibitor that is specifically and highly expressed in silk glands, which can significantly prevent the proliferation of gastric cancer cells by inhibiting the MAPK/ERK pathway initiated by EGF/EGFR through the promotion of expression of the proto-oncogene c-Myc, thereby affecting the expression of related cyclins. Through molecular docking and virtual screening of silkworm carboxypeptidase inhibitors and epidermal growth factor receptors, we identified a polypeptide that overlapped with existing small-molecule inhibitors of the receptor. In the present work, we explore the medicinal potential and application of silkworm carboxypeptidase inhibitors to promote the development of anti-tumor drugs from insect-derived substances.

## 1. Introduction

Gastric cancer, an important disease threatening human health, is a common malignant tumor of the digestive system with high mortality and poor prognosis [1,2]. The International Agency for Research on Cancer (IARC) demonstrated that the incidence of gastric cancer ranks fifth among malignant tumors and the mortality rate ranks third among all malignant tumors [3]. In 2020, the number of new cases worldwide was close to 1.09 million, accounting for 5.6% of all malignant tumors, and the number of deaths in that year was approximately 769,000, accounting for 7.7% of all malignant tumors [4]. Research predicts that the global cancer burden will continue to increase by 2040, with a 47% rise from 2020 [5]. It is crucial, therefore, to explore the pathogenesis of gastric cancer and develop measures to prevent it.

Despite some progress in the application of systemic chemotherapy, radiotherapy, surgery, targeted therapy, immunotherapy, and other methods in the treatment of gastric cancer, the prognosis of gastric cancer patients is still relatively poor because the symptoms of gastric cancer usually only appear in the later stage [6,7]. Current chemotherapeutic drugs are often accompanied by serious side effects and additional pain for patients. Therefore, it is of great significance to screen and develop new anticancer drugs and treatment strategies with low toxicity and high efficiency [8]. The difluorinated prodrug {4-[bis(2-bromoethyl)amino]-3,5-difluorobenzoyl}-L-glutamic acid is activated by carboxypeptidase G2 showing good in vivo antitumor activity [9].

Carboxypeptidase inhibitors are a class of compounds with great potential for use in a variety of applications and have been identified in potatoes [10], ticks [11], roundworms [12], wolfberry [13], and many other species. For example, potato carboxypeptidase inhibitor, which contains 39 amino acid residues, is one such potential development candidate for agricultural applications [10]. Inhibition of fungal pathogens in rice was accomplished after the potato carboxypeptidase inhibitor was expressed in rice [14]. Tick carboxypeptidase inhibitor, a 75-amino acid peptide rich in cysteine, was found to bind tightly to plasma carboxypeptidase B and shows potential for prevention or treatment thrombosis [15]. Recently, the carboxypeptidase inhibitor β-lybatide has been discovered in wolfberry, in which it promotes antioxidant biological activity, and is the first and smallest highly stable plant carboxypeptidase inhibitor found in functional food [13].

In 2005, researchers first identified the carboxypeptidase gene in the silkworm epidermis and wing primordia [16]. The authors speculated that it was involved in the digestion of the old epidermis, thus allowing the silkworm to circulate amino acids more efficiently in their bodies. In previous studies, we comprehensively and systematically identified *Bombyx mori* carboxypeptidases by analyzing the data of the *Bombyx mori* genome database [17]. A total of 48 carboxypeptidases have been identified. Carboxypeptidases are expressed in different parts of *Bombyx mori* and involved in various functions. We found that carboxypeptidase specifically expressed in the midgut can participate in food digestion and nutrient utilization; however, carboxypeptidase inhibitors have never been reported in silkworms.

Epidermal growth factor receptor (EGFR) comprises a broad family of proteins whose members include EGFR (Erb B1/HER-1), HER-2 (Erb B2), HER-3 (Erb B3), and HER-4 (Erb B4) [18]. The EGFR family is distributed in the epithelial cell membrane and plays an important role in physiological processes such as cell growth, proliferation, and differentiation. EGFR is a transmembrane protein receptor with tyrosine kinase activity and can be structurally divided into three parts: the extracellular ligand binding domain, the transmembrane domain, and the intracellular kinase domain. Ligand-bound EGFR forms dimers and then autophosphorylates, activates tyrosine kinase receptors, and initiates downstream cascades to trigger signal transduction and activate several pathways such as the Ras/Raf/MEK/ERK-MAPK and PI3K/Akt/mTOR pathways, and ultimately regulates related transcription factors and participates in cell proliferation. Therefore, EGFRs have an important relationship with the development of tumor cells [19].

EGFR is highly expressed in 40–60% of gastric cancer cells and targeting EGFR is a current therapeutic strategy for preventing the development of gastric cancer [20,21]. At present, the existing targeted drugs against the EGFR family are mainly divided into two categories: monoclonal antibodies (mAbs) acting on the extracellular domain and small molecule inhibitors acting on the intracellular domain. Cetuximab affects the growth of tumor cells by inactivating the receptor by competitive binding [22]. Lapatinib inhibits tyrosine kinase phosphorylation and blocks signal transduction [23]. Trastuzumab can significantly prolong the survival of HER-2-positive gastric cancer patients with reduced toxicity and side effects [24]. Trastuzumab is currently the only monoclonal antibody that has been validated for human use through phase III clinical studies and was approved by China as an HER-2-positive drug in 2012. However, in Chinese patients with advanced gastric cancer, the HER2-positive rate is only 12–13%, so the application of trastuzumab is still relatively limited [25]. Other mAbs and EGFR tyrosine kinase inhibitors alone do not show significant advantages in the treatment of advanced gastric cancer. Therefore, it is particularly important to further explore anti-EGFR family-targeted drugs and molecular-targeted therapy for gastric cancer treatment.

In this study, we found that the silkworm carboxypeptidase inhibitor is highly expressed in the silkworm silk gland, which can inhibit the expression of proto-oncogene c-Myc and significantly inhibit the proliferation of gastric cancer cells. Based on the anticancer activity of the silkworm carboxypeptidase inhibitor, we designed the effective peptide of the silkworm carboxypeptidase inhibitor as a leading compound for tumor therapy, and found that it has potential as a drug for gastric cancer.

## 2. Results

### 2.1. Subsection Isolation and Activity Evaluation of Bombyx mori Recombinant Carboxypeptidase Inhibitor

**Figure 1:**
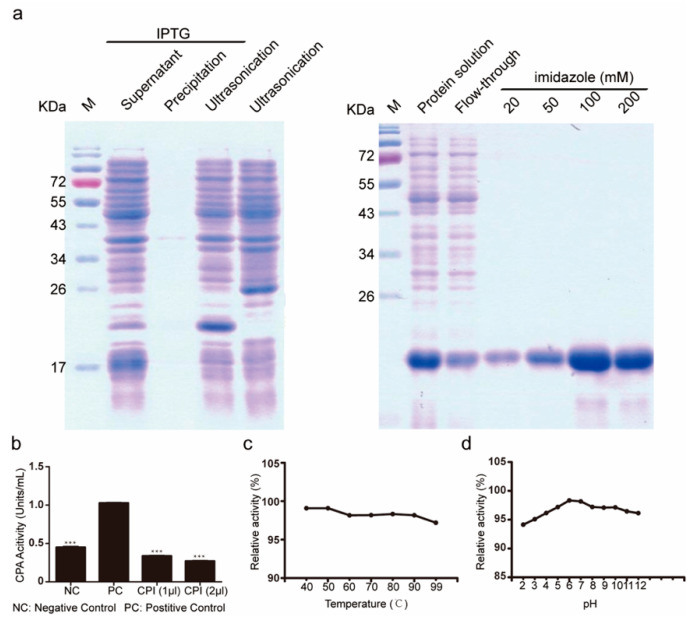
The expression and activity evaluation of recombinant silkworm carboxypeptidase inhibitor. (**a**) Prokaryotic expression, isolation, and purification of silkworm carboxypeptidase inhibitor protein. (**b**) Determination of the inhibitory activity of recombinant silkworm carboxypeptidase inhibitor. (**c**) Thermostability assay of recombinant silkworm carboxypeptidase inhibitors. (**d**) Acid–base stability assay of the recombinant silkworm carboxypeptidase inhibitor. PC means positive control potato carboxypeptidase inhibitor; NC means negative control PBS. The differences between the experimental and the control groups were analyzed by the Student’s *t* test, *** *p* < 0.001.

**Figure 2:**
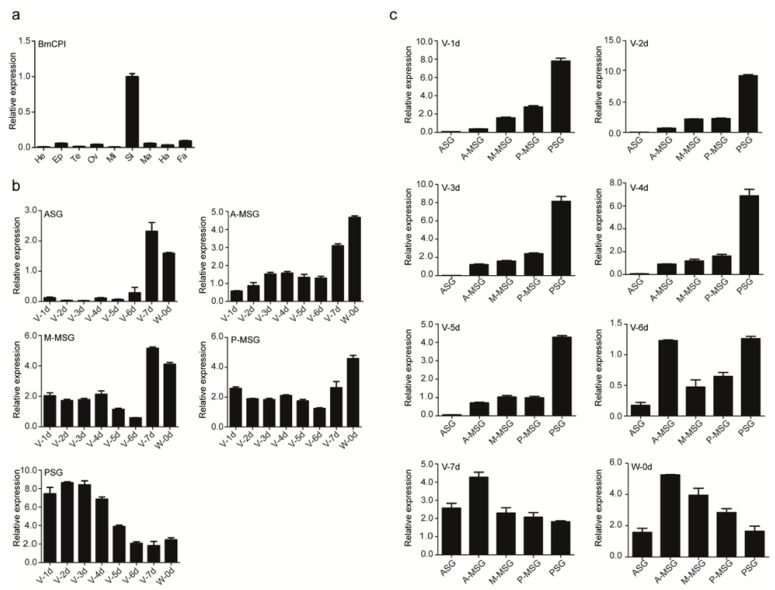
*Bombyx mori* carboxypeptidase inhibitors are specifically and highly expressed in silk glands. (**a**) Quantitative PCR results showed that the carboxypeptidase inhibitor was specifically and highly expressed in silk glands of *Bombyx mori*. (**b**) Expression of carboxypeptidase inhibitors in each segment of silkworm fifth instar silk gland. (**c**) Expression of carboxypeptidase inhibitors in silk glands at different stages (early and late) of fifth instar silkworms. Abbreviations: He, head; Ep, epidermis; Te, testis; Ov, ovary; Mi, midgut; Si, silk gland; Ma, Malpighian tube; Ha, hemocytes; Fa, fat body; ASG, anterior silk gland; A-MSG, anterior area of the middle silk gland; M-MSG, middle area of the middle silk gland; P-MSG, posterior area of the middle silk gland; PSG, posterior silk gland, V-1d, first day of the fifth instar; W-0d, the mounting.

The *Bombyx mori* carboxypeptidase inhibitor was prokaryotically expressed using pet28a as a vector. The peptide was abundantly secreted in the supernatant and isolated using an imidazole gradient, eluted at 100 mM and 200 mM imidazole (Figure 1a). To verify that the recombinant silkworm carboxypeptidase inhibitor was active, we carried out a carboxypeptidase activity inhibition experiment using potato carboxypeptidase. Our results showed that the recombinant silkworm carboxypeptidase inhibitor and potato carboxypeptidase inhibitor had similar inhibitory activity on potato carboxypeptidase (Figure 1b). To determine the inhibitory stability of the silkworm carboxypeptidase inhibitor, the peptide was tested under different temperature and pH conditions, followed by measurements of inhibitory efficiency directed towards silkworm carboxypeptidase activity (Figure 1c,d). Thermal stability analysis showed that with the increase of treatment temperature, the inhibitory activity of silkworm carboxypeptidase inhibitors on carboxypeptidase decreased slightly, but continued to show high inhibitory efficiency, thus suggesting good thermostability. *Bombyx mori* carboxypeptidase inhibitor treatment was pH-stable in the range of pH 2–6 and the inhibitory efficiency gradually increased at pH 6–7, remaining stable up to pH 8 but diminishing in more alkaline conditions. We also examined the tissue-specific expression of *Bombyx mori* carboxypeptidase inhibitor in fifth-instar, three-day-old *Bombyx mori* by quantitative PCR and found that it was highly expressed in the silk glands (Figure 2a). Quantitative PCR showed that the expression of carboxypeptidase inhibitor increased gradually from the anterior silk gland to the posterior silk gland in the middle and early stage of the fifth instar larvae. In the late fifth instar, the expression of carboxypeptidase inhibitor in the anterior and middle silk gland increased, and decreased in the posterior silk gland (Figure 2b). The expression pattern of carboxypeptidase inhibitors in the anterior silk gland and the anterior region of the central silk gland of the silkworm is similar. The expression amount is low in the early and middle stages. The expression amount increases from the seventh day of the fifth instar to the mounting, while the expression amount is relatively stable in the central and posterior regions of the central silk gland. In the posterior silk gland, the expression is high in the early middle stage, and low in the mounting (Figure 2c).

### 2.2. Evaluation of the Ability of Bombyx mori Carboxypeptidase Inhibitor Recombinant Protein to Inhibit the Proliferation of Gastric Cancer Tumors

**Figure 3:**
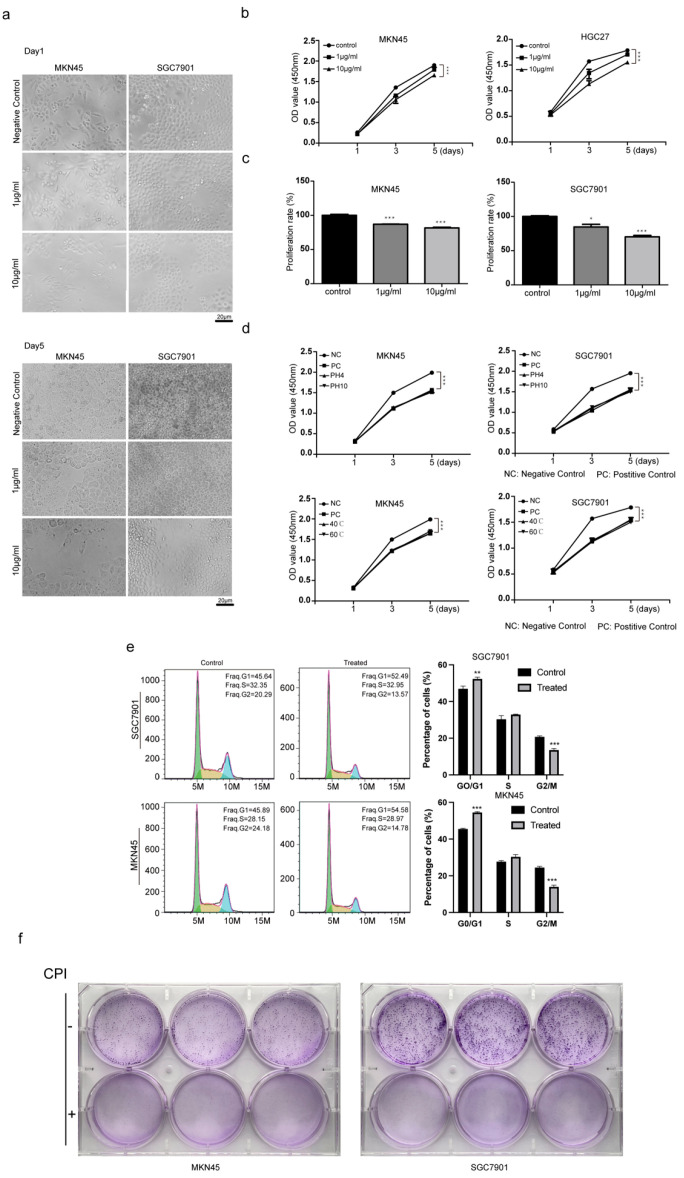
The recombinant protein of the silkworm carboxypeptidase inhibitor significantly inhibited the proliferation of gastric cancer cells. (**a**) Gastric cancer cells treated with silkworm carboxypeptidase inhibitor. (**b**) Proliferation of gastric cancer cells was measured by the CCK-8 method. (**c**) Inhibition rate of silkworm carboxypeptidase inhibitors on gastric cancer cells. (**d**) Potent *Bombyx mori* carboxypeptidase inhibitor inhibitory activity on gastric cancer cells under different conditions. (**e**) Cell cycle analyses after *Bombyx mori* carboxypeptidase inhibitor treatment. (**f**) *Bombyx mori* carboxypeptidase inhibitor inhibits colony formation in gastric cancer cells. The differences between the experimental and the control groups were analyzed by the Student’s *t* test, * *p* < 0.05, ** *p* < 0.01, *** *p* < 0.001.

According to the degree of tumor differentiation, we selected MKN45 cells with a moderate degree of differentiation and SGC7901 cells with a low degree of differentiation as experimental subjects. Cells in the logarithmic growth phase were cultured on 96-well plates with or without 1 μg/mL and 10 μg/mL carboxypeptidase inhibitor protein. On the first day, there was no significant difference in cell morphology between the groups, and cells were arranged loosely and uniformly. On the fifth day, cells were confluent in the negative control wells, while in the wells containing 10 μg/mL *Bombyx mori*, the carboxypeptidase inhibitor showed reduced cell proliferation (Figure 3a). Concurrently, a cell counting kit-8 (CCK-8) cell proliferation experiment was carried out, and the growth rate of the cells added with the silkworm carboxypeptidase inhibitor was slower than that of the negative control group, yet the growth time increases from the initial cell counts of the three treated cell lines were similar (Figure 3b). After the data were measured on the third and fifth days, a significance analysis was performed; it was found that the silkworm carboxypeptidase significantly inhibited the proliferation of gastric cancer cells. The cells on the third day were selected to calculate the proliferation rate. The proliferation rate of the 10 μg/mL silkworm carboxypeptidase inhibitor group was 75–80% that of the negative control group, indicating significant inhibition of proliferation of gastric cancer cells (Figure 3c). Considering the degree of malignancy of the three cell lines, the inhibitory effects of the silkworm carboxypeptidase inhibitor were greatest on moderately differentiated MKN45 cells and lowest on low-differentiated SGC7901 cells. Tumors exist in a complex in vivo environment. To investigate the inhibitory stability of the silkworm carboxypeptidase inhibitor on gastric cancer cells, we tested the silkworm carboxypeptidase inhibitor under different temperature and pH conditions, and subsequently measured its inhibitory effect on the proliferation of gastric cancer cells (Figure 3d). The experimental results showed that the treatment groups that were treated with silkworm carboxypeptidase inhibitors began to show significant differences compared to the negative control group by the third day of the treatment. Compared to the negative control group, those that were treated under different temperature and pH conditions maintained a good inhibitory effect on the proliferation of gastric cancer cells. After 3 days of treatment of gastric cancer cells with *Bombyx mori* carboxypeptidase inhibitor at a concentration of 10 μg/mL, we found by flow cytometry cell cycle analysis that *Bombyx mori* carboxypeptidase inhibitor was able to block gastric cancer cells in the G1 phase, and the percentage of G1 phase cells was elevated by approximately 8% after *Bombyx mori* carboxypeptidase inhibitor treatment (Figure 3e). Subsequent plate cloning experiments using SGC7901 and MKN45 showed that the number of plate clones formed in the experimental group was significantly reduced compared to that formed in the negative control group (Figure 3f).

### 2.3. Molecular Mechanism of Silkworm Carboxypeptidase Inhibitor Recombinant Protein in Inhibiting Gastric Cancer Tumor Proliferation

**Figure 4:**
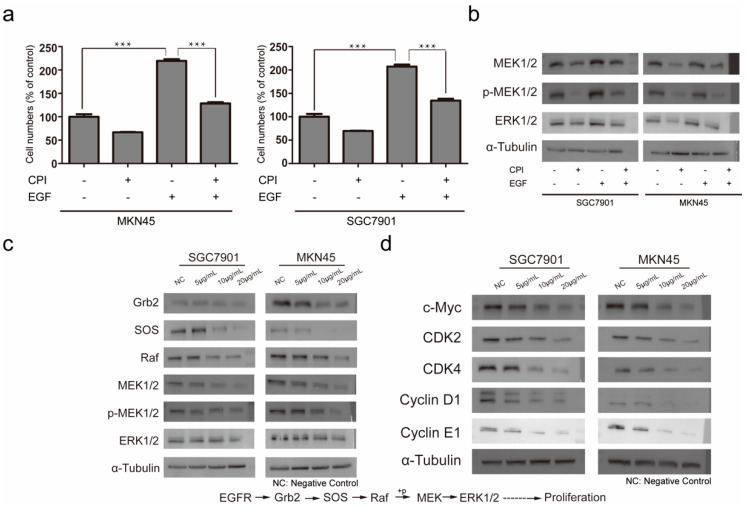
Exploration of the molecular mechanism of recombinant silkworm carboxypeptidase inhibitor protein inhibition of gastric cancer tumor proliferation. (**a**) The *Bombyx mori* carboxypeptidase inhibitor slows EGF-induced gastric cancer cell proliferation. (**b**) EGF stimulates MAPK/ERK pathway protein expression. (**c**) The *Bombyx mori* carboxypeptidase inhibitor affects MAPK/ERK pathway protein expression. (**d**) The *Bombyx mori* carboxypeptidase inhibitor affects the expression of c-Myc and its related cyclins. The differences between the experimental and the control groups were analyzed by the Student’s *t* test, *** *p* < 0.001.

EGF can promote the activation of EGFR, thereby promoting the rapid proliferation of tumor cells. After adding EGF, MKN45 and SGC7901 both showed rapid proliferation. After adding EGF and silkworm carboxypeptidase inhibitor at the same time, the proliferation rate was was significantly less than that in the EGF-only group (Figure 4a). Western blot results showed that EGF could activate MAPK/ERK pathway proteins. However, the expression of the MAPK/ERK pathway protein was affected by the addition of silkworm carboxypeptidase inhibitor (Figure 4b). We measured the expression of related proteins upstream of the MAPK/ERK pathway and found that the expression of growth factor receptor binding protein 2 (Grb2), guanylate exchange factor (SOS) and Raf protein, the key protein of the cascade reaction, were down-regulated by the addition of *Bombyx mori* carboxypeptidase inhibitor in a dose-dependent manner (Figure 4c). Raf protein is highly correlated with the phosphorylation of MEK1/2 and the down-regulated expression of Raf also strongly suggests that the silkworm carboxypeptidase inhibitor may be related to MAPK/ERK pathway. MEK1/2 and ERK1/2, which are related to the MAPK/ERK pathway, were down-regulated and MEK1/2 phosphorylation was inhibited, also in a dose-dependent manner. According to the signal pathway map, we detected the expression of the proto-oncogene c-Myc and the down-regulated expression of MAPK/ERK pathway-related proteins, which led to the down-regulated expression of the c-Myc protein. The down-regulated expression of c-Myc can inhibit the expression of cyclins such as CDK2, CDK4, Cyclin D1, and Cyclin E1, finally leading to the inhibition of cell proliferation (Figure 4d). Through the above experiments, it was found that the silkworm carboxypeptidase inhibitor could downregulate the expression of c-Myc.

### 2.4. Screening of Lead Compounds for Gastric Cancer Based on Effective Peptides of Bombyx mori Carboxypeptidase Inhibitor Recombinant Protein

**Figure 5:**
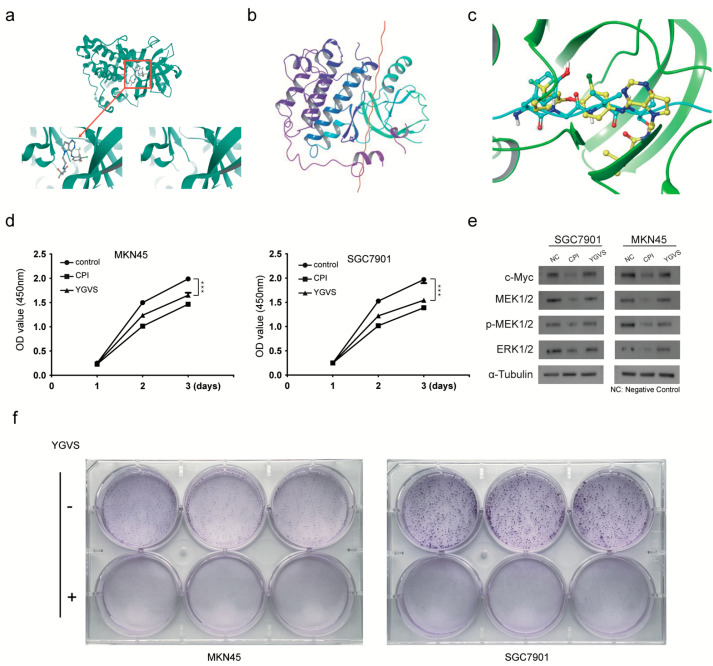
Screening of the lead compounds for the treatment of gastric cancer based on the effective peptide fragments of the *Bombyx mori* carboxypeptidase inhibitor recombinant protein. (**a**) Successful removal of the original small molecule ligand of the receptor. (**b**) Molecular docking of the silkworm carboxypeptidase inhibitor with the EGFR kinase domain. (**c**) The action site of the silkworm carboxypeptidase inhibitor peptide overlaps with the small molecule inhibitor tak-285. (**d**) The effective polypeptide of the silkworm carboxypeptidase inhibitor significantly inhibited the proliferation of gastric cancer cells. (**e**) Effective polypeptides of silkworm carboxypeptidase inhibitors affect the expression of MAPK/ERK pathway proteins. (**f**) Effective *Bombyx mori* carboxypeptidase inhibitor polypeptides inhibit gastric cancer cell colony formation. The differences between the experimental and the control groups were analyzed by the Student’s *t* test, *** *p* < 0.001.

Using PyMOL software, the original ligand on the receptor 3poz protein structure, namely, the small molecule tak-285, was successfully removed (Figure 5a). The ligand *Bombyx mori* carboxypeptidase inhibitor and the modified receptor EGFR kinase domain were then molecularly docked (Figure 5b). After 300,000 simulated dockings, the system returned 30 possible docking modes with the highest scores and selected the first-ranked docking mode for further analysis. In the ligand-active pocket, the *Bombyx mori* carboxypeptidase inhibitor peptide tyrosine–glycine–valine–serine (YGVS) overlapped with the small molecule inhibitor tak-285, suggesting that this peptide may inhibit the epidermis (Figure 5c). To test whether the effective peptide segment of silkworm carboxypeptidase inhibitor has the potential to become a lead compound, we conducted a CCK-8 experiment and found that, although the inhibitory ability of the effective peptide segment was slightly weaker than that of the silkworm carboxypeptidase inhibitor, it could still significantly inhibit the proliferation of gastric cancer cells (Figure 5d). The detection of MAPK/ERK pathway protein expression showed that the effective peptide segment could down-regulate the expression of MEK1/2 and ERK1/2 (Figure 5e). The plate cloning experiment found that compared with the clones formed by the control cells, the cell clones were significantly smaller, and the cell number decreased after adding the effective polypeptide fragment of the silkworm carboxypeptidase inhibitor (Figure 5f).

## 3. Discussion

Although invertebrates have a large number of protease inhibitors, research on protease inhibitors is mainly concentrated in vertebrates. Carboxypeptidase inhibitors, as a class of many protease inhibitors, have more research reports in invertebrates. The majority of studies on the protease inhibitors of silkworms are mostly focused on serine protease inhibitors. A study found that at least 19 silkworm serine protease inhibitors were abnormally expressed after microbial infection of silkworms and these serine protease inhibitors were predicted to be involved in immune processes [26]. *Bombyx mori* carboxypeptidase inhibitors have not yet been reported, and their relevant tissue-specific functions are still unclear.

In this paper, a carboxypeptidase inhibitor was reported for the first time in the silkworm, which not only exists in the silkworm but is also one of the components of silk. In our prokaryotic expression experiment, the silkworm carboxypeptidase inhibitor was expressed in the supernatant, which provided convenience for purifying and developing an antibody for measuring its expression. In *Bombyx mori*, carboxypeptidase inhibitors are highly expressed in silk glands. At the fifth instar stage of silkworms, characterized by the intense activity of silk gland proteins synthesis, the expression pattern of silkworm carboxypeptidase inhibitors gradually transitioned from high expression in the posterior silk glands in the early fifth instar to high expression in the middle and anterior silk glands in the late fifth instar, consistent with the proteomic pattern reported in previous studies [27]. Some studies have pointed out that the silkworm trypsin inhibitor CSTI is highly expressed in the silk glands of the fifth instar and can be detected in the cocoon net, similar to our measurement of the carboxypeptidase inhibitor. This expression assists in the proper formation of the silk fibers [28]. Two protease inhibitors, SPI1 and SPI2, were found in the cocoon silk of *Galleria Mellonella*; these inhibit the activity of exogenous proteases that cause damage to the body, inhibit the self-destruction of microorganisms, and protect the normal development of pupae [29]. A large number of protease inhibitors are highly expressed in silk glands and secreted into silk to perform various functions such as aiding the formation of silk fibers and possessing antibacterial properties. *Bombyx mori* carboxypeptidase inhibitors may also play a similar role.

We found that *Bombyx mori* carboxypeptidase inhibitor efficiency is very stable after different temperature and pH treatments. This excellent stability may be related to its cysteine-rich structure. Cysteine can generally form a stable structure, and low-molecular-weight protease inhibitors rich in cysteine are often very stable. Studies have reported that the silkworm serine protease inhibitors SPI38 and SPI39 are rich in cysteine and have good thermal and acid–base stability [30]. Their excellent thermal and acid–base stability is very helpful for further exploring the scientific value and practical application of silkworm carboxypeptidase inhibitors.

Gastric cancer is a common digestive system disease with a high degree of malignancy. The current mainstream treatment methods are still surgical resection, chemotherapy, and radiotherapy [31]. Similar to other tumors, chemotherapy resistance and other problems are encountered during the treatment process. In addition, chemotherapy, radiotherapy, and other treatment methods have serious side effects. Therefore, it is of critical importance to find new drugs with low toxicity that can circumvent resistance. Antitumor bioactive proteins have many advantages over chemical drugs, such as excellent cell dispersibility, specificity for certain tumorigenic signals, and a lack of genotoxic effects.

To evaluate the ability of silkworm carboxypeptidase inhibitors to inhibit the proliferation of gastric cancer, we examined different concentrations of silkworm carboxypeptidase inhibitors and their ability to inhibit cell proliferation in several cancer cell lines of contrasting differentiation and proliferation potential. After five days of culture, cells in the experimental group were larger and the number of cells was fewer than that in the control group, thus suggesting that silkworm carboxypeptidase inhibitors may inhibit the proliferation of gastric cancer cells. To verify this assumption, we used the CCK-8 method to measure the cell growth curve and the results showed that the cell growth rate of the experimental group was significantly inhibited and showed a significant dose-dependence. The natural active ingredients contained in silkworms have been previously reported to have antitumor activity. The silkworm antimicrobial peptide cecropin-XJ can significantly inhibit the proliferation of esophageal cancer cells and induce their apoptosis when combined with chemotherapeutic drugs, while having less toxicity than normal cells [32]. The proliferation rate of the 10 μg/mL silkworm carboxypeptidase inhibitor group on the third day was 75–80% of the negative control group and showed significant gastric cancer cell proliferation inhibition. There were differences in effects based on the differentiation potential of cell lines examined, with moderate inhibitory effects in MKN45 and poor inhibitory effects on SGC7901 cells. Silkworm carboxypeptidase inhibitors prevent gastric cancer cells’ proliferation and have clinical relevance. Research shows that carboxypeptidases are present in the gastrointestinal tract as well as in the gastric cancer-related bacterium *Helicobacter pylori*, and are up-regulated in gastric carcinomas [33,34,35]. Another study found that the expression of an endogenous carboxypeptidase inhibitor is down-regulated in human gastric carcinoma [36]. The silkworm carboxypeptidase inhibitor may be able to compensate for the functional loss caused by the down-regulation of the endogenous carboxypeptidase inhibitor. Drug stability after administration is crucial for target delivery. Fluorouracil has always been the core drug used for the treatment of digestive tract tumors, but the main challenge facing the use of fluorouracil in the treatment of gastric cancer is its short half-life, which greatly affects its anti-cancer effect. Therefore, we tested the tumor inhibition of silkworm carboxypeptidase inhibitor under different temperatures and pH conditions to ascertain its stability and found the ability of the peptide to inhibit the proliferation of gastric cancer cells was almost unaffected regardless of temperature and pH conditions, showing stable gastric cancer cell inhibitory activity.

The signal transduction in gastric cancer cells is very complex with numerous active interacting pathways, including EGF, Wnt, TGFβ, and other ligands that activate cascade reactions and participate in cell cycle regulation, proliferation, differentiation, and other physiological processes [37,38,39]. Faced with a complex network of signaling pathways, it is very difficult to find the exact pathway inhibited by silkworm carboxypeptidase inhibitors. Previous studies have reported that potato carboxypeptidase inhibitors can inhibit the proliferation of pancreatic cancer by acting as a competitive inhibitor of epidermal growth factor (EGF) and by binding to EGFR [40]. Excessive activation of EGFR is associated with the occurrence and development of various epithelial cell cancers such as lung cancer and colon cancer [41,42]. Overactive EGFRs have also been detected in poor-prognosis, non-small cell lung cancer cases [43]. EGFR is closely linked to cancer development, making it a candidate target for cancer therapy. A likely target of *Bombyx mori* carboxypeptidase inhibitor inhibitory function may be EGFR. EGF is a heat-resistant, single-chain, low-molecular-weight polypeptide that can specifically recognize and bind to EGFR on target cells and initiate a series of biochemical reactions and finally promote DNA synthesis and the mitosis of target cells. We added EGF to stimulate cell proliferation in gastric cancer cells and found that the proliferation rate was significantly accelerated; yet, after the addition of silkworm carboxypeptidase inhibitor, the rapid increase in the number of gastric cancer cells was curbed, although the number of cells was still higher than that of the previous EGF-free treatments. The negative control group with EGF was significantly lower than the positive experimental group added with EGF only. When different concentrations of EGF were added to lung adenocarcinoma A549 cells, the protein expression level of EGFR increased to different degrees [44]. *Bombyx mori* carboxypeptidase inhibitor can inhibit the rapid proliferation of gastric cancer cells induced by the addition of EGF, which indicates that the inhibitory activity of silkworm carboxypeptidase inhibitor is likely related to the downstream-related pathways initiated by EGF/EGFR.

EGF promotes cell proliferation primarily by activating the downstream extracellular signal-regulated kinase (ERK) pathway through the phosphorylation of EGFR [45,46]. The expression of MEK1/2 and ERK1/2 proteins was up-regulated due to stimulation by EGF and down-regulated after the addition of *Bombyx mori* carboxypeptidase inhibitor. The pathway of *Bombyx mori* carboxypeptidase inhibitor in inhibiting gastric cancer is highly correlated with MAPK/ERK. When we examined the proteins in the MAPK/ERK pathway we found that, because of the addition of the silkworm carboxypeptidase inhibitor, the expression of related proteins in this pathway were down-regulated in a dose-dependent manner. The MAPK/ERK pathway is a common signaling pathway for tumor migration and invasion and plays an important role in the development of tumors. The target of icotinib is EGFR, which can inhibit its activity and block signaling. Compared with the lung cancer HCC827 cells in the control group, the icotinib treatment group inhibited the activation of the downstream MAPK/ERK signaling pathway by inhibiting the autophosphorylation of EGFR, and it also inhibited the proliferation of HCC827 cells [47]. After treatment with *Bombyx mori* carboxypeptidase inhibitor, the abnormal expression of MAPK/ERK indicated that this pathway is the key conduit for the inhibition of gastric cancer cell proliferation. The detection of tumor suppressor factors downstream of this signaling pathway showed that the expression of tumor suppressor c-Myc protein was down-regulated after being treated with the *Bombyx mori* carboxypeptidase inhibitor. Aberrant expression of c-Myc leads to genomic instability and tumorigenesis and maintains tumor growth [48]. One study found that the mRNA and protein expression of c-Myc were increased in HGC-27 and SGC-7901 gastric cancer cell lines compared with the human gastric mucosal cell line GES-1, and knockdown of c-Myc expression inhibited gastric cancer cell proliferation [49]. Therefore, the silkworm carboxypeptidase inhibitor likely down-regulated the expression of c-Myc, leading to an inhibited proliferation of gastric cancer cells. The expression of c-Myc-related cyclins was also detected and it was found that four cyclins, including CDK2, CDK4, Cyclin D1, and Cyclin E1, were down-regulated after treatment with *Bombyx mori* carboxypeptidase inhibitor. c-Myc can affect the cell cycle by regulating cyclin kinases, which can bind to the E-box on the CDK4 promoter and upregulate CDK4 expression [50]. However, the regulation of c-Myc on CDK2 is still unclear. There is no clear relationship between c-Myc and CDK2 in rat cells, but in gastric cancer cells, c-Myc can up-regulate the expression of CDK2 [51]. The relationship between c-Myc and Cyclin D1 varies greatly by species and cell line. In gastric cancer cells, c-Myc up-regulates Cyclin D1 expression, while in rabbit embryo cells, c-Myc down-regulates Cyclin D1 expression. In other species, c-Myc and Cyclin D1 are not clearly related [52]. c-Myc can also promote the complex formation of Cyclin E and CDK2 [53]. The relationship between c-Myc and cyclin is very close. *Bombyx mori* carboxypeptidase inhibitor causes an abnormal expression of c-Myc, which in turn leads to the expression of cyclin, resulting in a disorder of the cell growth process. The relationship between c-Myc and cyclin is very close. *Bombyx mori* carboxypeptidase inhibitors cause the abnormal expression of c-Myc, which in turn leads to the abnormal expression of cyclin, resulting in arrest of the cell cycle in the G1 phase and disruption of the growth process. We confirmed that the silkworm carboxypeptidase inhibitor inhibited the expression of c-Myc through the MAPK/ERK pathway initiated by EGF/EGFR, thereby affecting the related cyclins and inhibiting the proliferation of gastric cancer cells.

Currently, many scholars are focused on the discovery of lead compounds from active ingredients of natural products. Different sources of natural active ingredients can be divided into plant sources, animal sources, microbial sources, and marine biological sources. As novel agents, silkworm carboxypeptidase inhibitors inhibit the proliferation of gastric cancer cells through the MAPK/ERK pathway, indicating that silkworm carboxypeptidase inhibitors have the potential to be therapeutic drugs for gastric cancer. Therefore, we also used molecular docking virtual screening technology, CCK-8 cell proliferation experiments, and plate cloning to evaluate the peptide as potential gastric cancer lead compounds for further development.

COVID-19 infection is triggered by the binding of the spike protein (S protein) of the virus to angiotensin-converting enzyme-2 (ACE2). Genetic material (RNA), with the participation of 3CLpro, is successfully replicated to produce a new type of coronavirus [54]. The natural marine polysaccharide chitosan has the activity of inhibiting the SARS virus. Some studies have used molecular docking technology to screen the S protein, ACE2 blocker, and 3CLpro inhibitor from oligomeric chitosan to find potential inhibitors of COVID-19. Molecular docking provides new ideas and methods for the prevention and treatment of COVID-19 prior to benchwork verification [55]. Therefore, we adopted the method of molecular docking virtual screening for candidate peptides that may play a key inhibitory role in inhibiting the proliferation of gastric cancer cells. First, the original small molecule inhibitor of the receptor was removed from the active center of the receptor. After the receptor was processed, the carboxypeptidase inhibitor was introduced into the molecular docking experiment 300,000 times. After analyzing the results with the highest score, we found that in the receptor active pocket, some peptides of *Bombyx mori* carboxypeptidase inhibitors coincide with the positions of small molecule inhibitors. It is speculated that this peptide domain may play a key role in the process of inhibiting the tumor proliferation activity of *Bombyx mori* carboxypeptidase inhibitors. After obtaining the screening results, we synthesized partial polypeptide fragments to evaluate their anti-proliferation effect on gastric cancer. The results of CCK-8 experiments showed that the polypeptides could inhibit the gastric cancer cell linesMKN45 and SGC7901. Molecular cloning experiments also confirmed that the polypeptides could reduce the proliferation rate of gastric cancer cells, but their inhibitory ability was slightly weaker than that of native silkworm carboxypeptidase inhibitors. Key proteins in the MAPK/ERK pathway were detected and we determined that the polypeptides downregulate the expression of MEK1/2, ERK1/2, and c-Myc. The regulation of tumor cell proliferation by inhibitors is very complex and may not be limited to a single mechanism. Protease inhibitors exist in very stable structures and can help maintain anti-tumor activity to a certain extent.

The drug development process generally includes the discovery, optimization, and development of lead compounds [56]. Effective polypeptide lead compounds of *Bombyx mori* carboxypeptidase inhibitors as drug candidates need to be further optimized in many ways. Pharmacodynamics comprises a key factor for the function of a drug. The use of pharmacophore splicing, isosteric replacement, and other methods can further improve the silkworm carboxypeptidase inhibitor peptide fragment [57]. The anti-gastric cancer efficacy of enzyme inhibitor polypeptide fragments is improved by reducing the alert structure in the optimized polypeptide or by avoiding the production of active metabolites and can effectively reduce its potential drug toxicity, while the introduction of phosphate groups can improve the water solubility of drugs, regulate their physicochemical properties, and improve bioavailability [58]. Additional work towards enhancing the binding ability to the EGFR can further prolong peptide half-life, improve their pharmacokinetic properties, and precisely adjust the pharmaceutical properties to make them more suitable for clinical application [59].

In summary, we report the first *Bombyx mori* carboxypeptidase inhibitor that is specifically and highly expressed in silk glands. As a biologically active ingredient, it can inhibit the expression of the proto-oncogene c-Myc by affecting the MAPK/ERK pathway initiated by EGF/EGFR, thereby inhibiting the proliferation of gastric cancer cells. The abnormally high expression of c-Myc is a common feature of gastric cancer patients with poor prognosis. Based on the discovery that silkworm carboxypeptidase inhibitors can inhibit the expression of c-Myc, we designed effective peptides of silkworm carboxypeptidase inhibitors as tumor therapy lead compounds, explored the potential of silkworm carboxypeptidase inhibitors as gastric cancer drugs, and here provide clues for subsequent clinical applications.

## 4. Materials and Methods

### 4.1. Biological Materials

The silkworm strain Dazao (p50) was used in this study. The silkworms were reared on fresh mulberry leaves in an environment maintained at 25 °C with 70–80% relative humidity and a 16 h light/8 h dark cycle in the professional room of the State Key Laboratory of Silkworm Genome Biology. Samples from embryonic stages and larval tissues were isolated and stored in liquid nitrogen.

The gastric cancer cell lines SGC7901 and MKN45 used in this study were purchased from the American Type Culture Collection (ATCC). Cells were passaged and preserved by the Biology Research Center, Institute of Frontier Interdisciplinary Studies, Southwest University.

### 4.2. Western Blot

Protein samples were obtained and the protein concentration was determined by the BCA method for equal gel loading volumes. Sample constituents were separated by SDS-PAGE gel electrophoresis at 100 V for 40 min and blotted to a PVDF membrane as follows. Membranes were washed in methanol for 10 s, then transferred for 5 min to double distilled water, followed by placement in transfer solution. Band transfer to membranes was conducted at 260 mA for 1 h. After transfer, membranes were washed 2× with washing solution followed by 8 mL of QuickBlock™ Western Blocking Solution (Beyotime, Shanghai, China). Blots were blocked for 10 min at 25 °C on a platform shaker, then washed 2× in washing solution. Primary antibodies against C-myc (1:1000 abcam ab32072), MEK1/2 (1:1000 abcam ab32091), MEK1/2 (phospho S298) (1:1000 abcam ab96379), ERK1/2 (1:10,000 abcam ab184699), Grb2 (1:1000 abcam ab32111), SOS (1:1000 abcam ab140621), Raf (1:1000 abcam ab200653), CDK2 (1:1000 beyotime AF1063), CDK4 (1:300 beyotime AC251), Cyclin D1 (1:500 beyotime AF1183), and Cyclin E1 (1:1000 beyotime AF2491) were added and incubated at 4 °C overnight with shaking. Membranes were then washed 3× for 5 min each at room temperature. Secondary antibody was added. Blots were washed 3× at room temperature for 5 min each in washing solution. Equal volumes of ECL Chemical Developer A and B (Beyotime, Shanghai, China) were added for color development.

### 4.3. qRT-PCR

Quantitative RT-PCR was performed with SYBR^®^ Premix Ex Taq™ II (TaKaRa, Shiga-ken, Japan) using a Step-One-Plus™ Real-Time PCR system (Thermo, Waltham, MA, USA). The PCR conditions were: 94 °C for 30 s, followed by 40 cycles at 95 °C for 5 s, and 60 °C for 30 s. All cDNA samples were normalized using the housekeeping sw22934 gene as an internal control. Each expression assay was repeated at least three times. Relative gene expression level was determined by the 2^−ΔΔCT^ method.

### 4.4. Acid–Base and Temperature Stability Analysis of Recombinant Silkworm Carboxypeptidase Inhibitor

The recombinant *Bombyx mori* carboxypeptidase inhibitor was placed in 7 different temperature environments for 15 min: 40 °C, 50 °C, 60 °C, 70 °C, 80 °C, 90 °C, and 99 °C. The pH was adjusted with acid–base adjustment solutions HCl and NaOH to prepare 11 separate Britton-Robinson buffers with a pH of 2, 3, 4, 5, 6, 7, 8, 9, 10, 11, and 12.

The recombinant silkworm carboxypeptidase inhibitors were placed in Britton-Robinson buffers and incubated overnight at 25 °C prior to the assay.

We determined the carboxypeptidase activity of the recombinant silkworm carboxypeptidase inhibitor after treatment under different temperature and pH conditions using the carboxypeptidase A activity assay kit (Sigma-Aldrich, St. Louis, MO, USA), following the manufacturer’s instructions.

Single comparisons were performed by the Student’s t-test with α = 0.05 (GraphPad Software, version: 9.3.0; http://www.graphpad.com/quickcalcs/ttest1.cfm (accessed on 1 July 2022)).

### 4.5. Plate Cloning

When the cell density reached 70%,cultured cells in Petri plates with 5 mL of PBS were washed in a 37 °C water bath by gentle shaking. After washing, 1 mL of PBS was added containing 0.05% trypsin with shaking at 37 °C for 2 min. Digestion was stopped by the addition of 2 mL PBS. Cells were then gently pipetted repeatedly to form a single-cell suspension, then transferred to a 5 mL centrifuge tube, and centrifuged at 1300 rpm for 3 min. Supernatants were removed and cells were resuspended in 1 mL of 1640 complete medium. Following cell counts, a culture medium with cells was diluted to achieve cell counts of 1000 cells per well for a six-well culture plate in a final volume of 2 mL. Plates were incubated overnight in CO_2_ incubator (5% CO_2_, 37 °C) (Thermo, Waltham, MA, USA). After verifying adequate cells’ adherence, cells were treated with recombinant silkworm carboxypeptidase inhibitor at a final concentration of 10 mg/L and incubated in a CO_2_ incubator. Cells were observed every other day until the number of single cell clones reached approximately 50. The medium was vacuum-aspirated and cells were washed 3 times with PBS for 5 min each time. The PBS was vacuum-aspirated, and the solution was replaced with 1 mL of 4% paraformaldehyde and fixed at room temperature for 15 min. Paraformaldehyde solution was vacuum-aspirated and replaced with a crystal violet solution for staining with gentle shaking for 30 min at room temperature.

### 4.6. CCK-8

Cultured cells were trypsin-digested (see above) to form a cell suspension, counted, diluted to concentrations of 1000 cells/well for 96-well culture plates, and spread evenly on the well surface. Cells were cultured overnight in a CO_2_ incubator (5% CO_2_, 37 °C). Cells became adherent and grew stably by the second day. The original culture medium was removed and a complete medium containing recombinant carboxypeptidase inhibitor or negative control complete medium was added. Cells were observed daily and CCK-8 experiments (Beyotime, Shanghai, China) were performed by the manufacturer’s protocol on days 1, 3, and 5 after dosing.

### 4.7. Bioinformatics Analysis

The silkworm carboxypeptidase inhibitor signal peptide was predicted using SignaIP 4.1 (http://www.cbs.dtu.dk/services/SignalP/ (accessed on 1 May 2022)). Information such as molecular mass and the isoelectric point was predicted using the ProtParam (http://web.expasy.org/protparam/ (accessed on 1 May 2022)) website. Protein domains were analyzed with Pfam (http://pfam.xfam.org/search (accessed on 1 May 2022)).

### 4.8. Molecular Docking

Ligand and protein receptor file preparation and processing.

We obtained the structure of the EGFR kinase domain in complex with the tak-285 Crystal structure (PDB id: 3poz) from the Protein Data Bank in Europe, https://www.ebi.ac.uk/pdbe/ (accessed on 1 July 2022)). The receptors were hydrogenated, charged, and the amino acid deletion structure was perfected using the Protein Preparation Wizard Panel in the computer-aided drug design software SchrÖdinger. The structure of the ligand *Bombyx mori* carboxypeptidase inhibitor was drawn in SchrÖdinger and the 3D structure of the ligand was constructed using the LigPrep Panel.

(1) Protein–protein docking

Based on the prepared macromolecular ligand and protein structure files, the Protein–Protein Docking panel in Bioluminate software was used for molecular docking, with the EGFR kinase region defined as the receptor and the silkworm carboxypeptidase inhibitor defined as the ligand body. Other parameters kept their default settings.

(2) Small molecule–protein docking

Based on the prepared protein structure file, the Receptor Grid Generation in SchrÖdinger was used to define the binding site with the following parameter settings: Center: Centroid of Workspace ligand. Molecular docking was then performed using the Ligand Docking panel using the default parameter settings.

Autodock_vina 1.1.2 was used to calculate the minimum binding energies between key EGFR and *Bombyx mori* carboxypeptidase inhibitors.

The evaluation of the binding mode generally refers to the binding energy, and the lower the binding energy, the better the binding mode. A binding energy of <0 kcal/mol is considered to indicate spontaneous binding between ligands and receptors. Binding energy is highly correlated with ligand–receptor affinity.

## Data Availability

All data generated for this research are included in the article.
